# Iron Supply Affects Anthocyanin Content and Related Gene Expression in Berries of *Vitis vinifera* cv. Cabernet Sauvignon

**DOI:** 10.3390/molecules22020283

**Published:** 2017-02-14

**Authors:** Pengbao Shi, Bing Li, Haiju Chen, Changzheng Song, Jiangfei Meng, Zhumei Xi, Zhenwen Zhang

**Affiliations:** 1 College of Enology, Northwest A&F University, Yangling 712100, Shaanxi, China; pengbaoshi@163.com (P.S.); 18700807714@163.com (B.L.); scz1103@gmail.com (C.S.); mjfwine@nwafu.edu.cn (J.M.); xizhumei@nwafu.edu.cn (Z.X.); 2 College of Food Science and Technology, Hebei Normal University of Science & Technology, Qinhuangdao 066600, Hebei, China; 3 College of Horticulture Science and Technology, Hebei Normal University of Science & Technology, Qinhuangdao 066600, Hebei, China; haijuchen@163.com (H.C.); 4 Shaanxi Engineering Research Center for Viti-Viniculture, Yangling 712100, Shaanxi, China

**Keywords:** grape berry, anthocyanins, gene expression, sugar, iron

## Abstract

Anthocyanins are important compounds for red grape and red wine quality, and can be influenced by supply of nutrients such as nitrogen, phosphorus, potassium, zinc, and iron. The present work aims to gain a better understanding of the effect of iron supply on anthocyanins concentration in grape berries. To this end, own-rooted four-year-old Cabernet Sauvignon grapevines (*Vitis vinifera*) were fertigated every three days with 0, 23, 46, 92, and 184 μM iron (Fe) from ferric ethylenediamine di (*o*-hydroxyphenylacetic) acid (Fe-EDDHA) in a complete nutrient solution. Fe deficiency or excess generally led to higher concentrations of titratable acidity and skin/berry ratio, and to lower reducing sugar content, sugar/acid ratio, pH, berry weight, and concentration of anthocyanins. Most of the individual anthocyanins detected in this study, except cyanidin-3-*O*-glucoside, delphinidin-3-*O*-glucoside, and cyanidin-3-*O*-(6-*O*-coumaryl)-glucoside, in moderate Fe treatment (46 μM) grapes were significantly higher than those of other treatments. Genes encoding chalcone isomerase (CHI), flavanone 3-hydroxylase (F3H), leucoanthocyanidin dioxygenase (LDOX), and anthocyanin *O*-methyltransferase (AOMT) exhibited higher transcript levels in berries from plants cultivated with 46 μM Fe compared to the ones cultivated with other Fe concentrations. We suggest that grape sugar content, anthocyanins content, and transcriptions of genes involved in anthocyanin biosynthesis were correlated with Fe supply concentrations.

## 1. Introduction

Iron (Fe) is an essential element for the growth and reproduction of plants, animals, and humans [1], and plays an important role in cell metabolism. In plants, morphological and physiological changes can be caused by Fe deficiency or excess [2,3]. Fe is a constituent of chlorophyll (Chl) synthesis, mitochondrial and photosynthetic electron transport chains, and regulation of Calvin cycle enzymes [2,4,5,6,7], such as ribulose-1,5-bisphosphate carboxylase/oxygenase (Rubisco), NADP-glyceraldehyde-3-phosphate dehydrogenase (GAPDH), phosphoribulokinase (PRK), stromal fructose-1,6-bisphosphatase (FBPase), and cytosolic FBPase [4]. Therefore, in Fe-deficient plants, many important physiological functions of Fe cannot operate normally, and plant growth is adversely affected.

The most obvious visible symptom of Fe-deficient plants is leaf chlorosis, owing to low content of Chls and carotenoids [8,9]. Iron chlorosis leads to reduced photosynthetic efficiency and electron transport, and hence carbon fixation [2,10]. Consequently, Fe deficiency influences root, shoot, and leaf growth and causes fruit yield decreases [1,2,10]. Numerous studies have investigated the effect of Fe on fruit quality. Most of the studies have focused mainly on the effect of Fe on fruit size, firmness, the concentrations of sugar, acidity, vitamin C, dry matter, and phenolic compounds [2,10,11,12,13,14]. Among the studied chemical compositions, anthocyanins are very important for fruit color and are involved in many biological functions in plants such as antioxidant capacity, protective effects against UV irradiation, and pathogen attack [15,16,17]. Hence, fully understanding the influence of Fe on the anthocyanin biosynthesis is important for optimizing anthocyanin content in fruits.

In grapes, anthocyanins are accumulated in red berry skins and in the pulp of ‘teinturier’ cultivars [17,18,19]. The biosynthesis of anthocyanins are through the flavonoid pathway, starting with phenylalanine as a precursor. The accumulation and the proportion of individual anthocyanins in the berry skin or cultured grape cells can be influenced by several mineral nutrients [17,20,21,22,23] including iron [14]. The molecular regulations of anthocyanin biosynthesis in response to mineral nutrients—such as nitrogen [17], phosphorus [22], and zinc [23]—have been studied. However, the effects of Fe on the anthocyanins have been little studied. Ahmed et al. reported that Fe supply increased the total anthocyanins of ‘Banaty’ grapes [14], furthermore, it has not yet been elucidated how individual anthocyanins and their biosynthesis pathway genes respond to different concentrations of Fe.

The current work was designed to study the effects of variable Fe levels on grape berry anthocyanin biosynthesis under greenhouse conditions. Special emphasis was put on its impact on the individual anthocyanin concentrations and expression levels of structural genes of the flavonoid pathway.

## 2. Results and Discussion

### 2.1. Effect of Different Iron Levels on Berry Physical and Chemical Characteristics

The effect of five iron concentrations (T0: 0, T1: 23, T2: 46, T3: 92, and T4: 184 μM) on berry physical and chemical characteristics are displayed in Table 1. Iron significantly influenced the sugar and acid contents of grape berries. Grape berries on T2 treatment accumulated obviously high reducing sugar (RS) but low titratable acid content (TAC), and hence a significantly increased (*p* < 0.001) RS/TAC ratio compared to other treatments. Both decreasing and increasing the Fe concentration significantly diminished (*p* < 0.001) TS but increased (*p* < 0.001) TAC. The trends of pH of different treatments were similar with RS. Similar berry weights were detected in the T0, T1, and T3 treatment, a high berry weight was detected in the T2, while T4 significantly decreased (*p* = 0.0021, 0.0231, 0.0003, and 0.0135 for T0, T1, T2, and T3, respectively) berry weight compared to other treatments (Table 1). In contrast, the vines of T2 treatment produced the lowest skin/berry ratio, but the difference between treatments was insignificant.

Content of sugars and acids greatly determines the organoleptic quality of grapes and the ratio of sugars to titratable acids is commonly used to evaluate fruit maturity [24]. In the present study, Fe deficiency and excess lead to poor wine grape quality, decreased RS content and increased TAC, and hence the lower RS/TAC ratio. Besides, the decreases in RS/TAC ratio suggest the delayed ripening of the berries of iron deficient and excess treatments; similarly, the retarded ripening caused by Fe deficiency was observed in peach [10,11] and citrus [25].

Numerous studies have described the enhancing effects of Fe treatments on the content of TSS or sugar in mature fruits [11,14]. In Fe-deficient plants, the activity of ribulose-1,5-bisphosphate carboxylase (RuBPC) and the content of Chl and carotenoids decrease, hence leaf CO_2_ exchange rate and Chl photosynthetic efficiency were decreased [2,4,8]. Iron excess also occurs in plants where it elicits an oxidative stress leading to necrotic spots in the leaves [5]. These might be the reasons that Fe appropriate treatment (T2) results in higher sugar content than Fe deficiency and excess treatments in this study.

Tartaric and malic acid are major organic acids in grape berry. The T2 treatment reduced the TAC in Cabernet Sauvignon fruits. This may be attributed to the decline of malic acid during fruit ripening. Being a major substrate of respiration, the malic acid in the berries of T2 treatment, which may have a higher leaf net photosynthetic rate, could be more broken down than other treatments to the CO_2_, and the latter was released to take part in the light reactions of photosynthesis [2]. Besides, Mark Kliewer et al. [26] reported that tartaric and malic acids decrease sharply after veraison and continue to decrease even after the fruit is ripe, so delays in maturity caused by Fe deficiency and excess could explain the higher TAC found in grape berries from Fe-deficient and excess trees when compared to T2 treatment. On the contrary, the pH of T2 grapes was higher than other treatments. Grapes with high pH levels need to be submit to an acidification process before alcoholic fermentation. According to the results for T1, T3, and especially for T2, the iron supply induced an increase of sugar content. However, we also detected negative results concerning grape acidity.

Berry weight, determined by berry size and density, is one of the essential factors contributing to grape quality [23]. The maximum berry weight of Cabernet Sauvignon was obtained with the T2 treatment. The increase may be due to enhanced synthesis of metabolites, increased absorption of water, and mobilization of sugars and minerals in the expended cells and intercellular spaces of the mesocarp. Fe deficiency reduced the activities of Rubisco and other photosynthetic enzymes, the Fe deficient grapevines have lower concentrations of nonstructural carbohydrates in source leaves and, therefore, are source limited [4].

### 2.2. Effect of Different Iron Levels on Berry Anthocyanin Composition

Typical chromatogram of skin extracts of Cabernet Sauvignon recorded at 525 nm, are displayed in Supplementary Materials Figure S1. The retention times, molecular ions, important fragment ions, tentative identification, and abbreviations of the anthocyanins quantified in the present study are given in Table 2. A total of 19 anthocyanins were found from the grape skins of different treatments. These compounds have been identified by their HPLC retention times, elution order, and fragmentation pattern. The identity and quantity of anthocyanins reported in the present study were in general accordance with previous findings in grapes [27,28,29,30].

**Figure 1:**
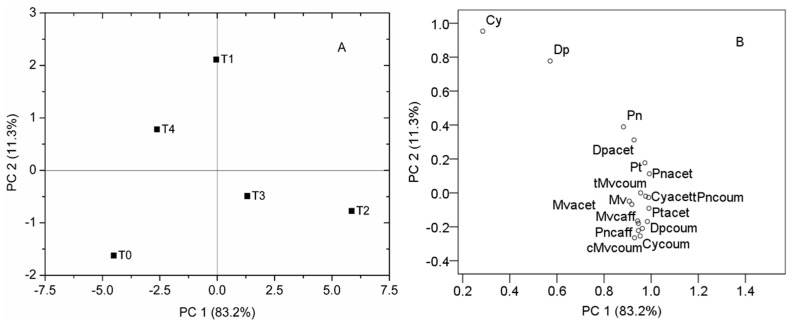
Principal component analysis (PCA) based on the correlation matrix of anthocyanins. Score plot (**A**) and loading plot (**B**). Abbreviations used in plot (**B**) are shown in Table 2.

The different Fe treatments had significant (*p* < 0.05) influence on the content and proportion of all anthocyanins quantified in the Cabernet Sauvignon grape berries (Table 3). Principal component analysis (PCA) was applied to visualize the general differences in berry content of anthocyanins among the different Fe treatments (Figure 1A,B). The first two principal components (PC) represented 83.2% and 11.3% of the variance, respectively, with a total variance of 94.5%. PC1 was obviously connected with the content of most individual anthocyanins except for Cy and Dp. Whereas, PC2 was decided by Cy and Dp. The differentiation between grapes of treatments was observed (Figure 1A). In the score plot, the anthocyanins of T2 treatment grapes are well separated along the first PC (83.2% of total variance) from the anthocyanins of T0 treatment grapes, then T4 treatment grapes. This suggests that the more severe Fe deficiency (T0) or excess (T4), the higher difference compared to Fe appropriate supply (T2) (see Table 3). Moreover, the anthocyanins of T0 treatment grapes are well separated from those of the T1 treatment along the second PC (11.3% of total variance). This means that slight Fe deficiency is beneficial to enhance Cy and Dp. The loading plot (Figure 1B) gives information about the relationships between the investigated anthocyanins. Compounds that are close to each other on the plot denote a strong positive correlation, while a strong negative correlation is denoted by compounds that are symmetrically distant on the loading plot area [33].

Analysis of anthocyanin composition clearly showed significant differences between the five different Fe concentration treatments. Compared to the T2 treatment berries, 58.9, 28.0, 9.2, and 53.2% decreases were found in the total anthocyanin concentration of the berries from treatments of T0, T1, T3, and T4, respectively (Table 3), and significant differences were observed between the five treatments (*p* < 0.05). The most abundant anthocyanins in grape berries in the present study were Mv, followed by the Mv-ac, tMv-cou, and Pn (Table 3). Additionally, the Fe deficiency and excess treatments changed the content of individual anthocyanins in the Cabernet Sauvignon berries. The T1 treatment increased the contents of precursors of anthocyanin (Cy and Dp), but decreased other individual anthocyanins compared to T2, and the content of almost all individual anthocyanins except Cy and Cy-coum was always significantly lower in T0, T3, and T4 treatments compared to T2 treatment. Appropriate Fe supply (T2) increased the levels of all forms of Pt, Pn, and Mv derivatives significantly.

As crucial pigments in red grapes, anthocyanins are fundamentally responsible for the red color of grapes and wines [34]. The proportion and amount of each anthocyanidin is influenced greatly by cultivar type and viticultural conditions. Among the many viticultural factors, fertilizer such as nitrogen [17,20], phosphate [22], potassium [21], zinc [23], iron [14], and so on are very important factors which can influence anthocyanin biosynthesis. Low nitrogen supply caused a significant increase in anthocyanin levels [17], higher nitrogen levels significantly decreased the anthocyanin concentration in the skin of berries at harvest [21]. The anthocyanin content at veraison increased in proportion to the potassium dose applied, however, the potassium level did not affect the must color density at harvest [21]. Foliage sprayed zinc sulfate enhanced the accumulation of total soluble solids, total phenols, flavonoids, flavanols, tannins, and anthocyanins in berry skin, decreasing the concentration of titratable acidity [23]. Chelated iron has been reported to enhance the accumulation of total anthocyanins in ‘Banaty’ grapes [14]. In our study, individual and total anthocyanin contents in berries were significantly higher in moderate Fe treatment (T2) than in Fe deficiency (T0, T1) and Fe excess (T3, T4) treatments, except Cy, Cy-coum, and Dp (Table 3). Due to the fact that glucose, fructose, and sucrose could induce anthocyanin accumulation in grape berries [18], the improvement of suitable Fe treatment on photosynthesis efficiency and sugar accumulation [2,4] could possibly enhance the biosynthesis of anthocyanins. Interestingly, Arozarena et al. [35] reported that high quality Tempranillo and Cabernet Sauvignon grapes had higher anthocyanin content than that of the low-quality ones, but in Garnacha grapes the obtained result is the opposite. This result suggested that the cultivar specificity is very important for anthocyanin profile of grapes, and could possibly explain that the lower quality grapes under severe Fe deficiency and excess condition have the lower anthocyanin content. Cy and Dp are considered to be the precursors for Pn and Mv and Pt, respectively [34]. The anthocyanins with more methoxyl groups in the B rings can contribute more redness [36]. The methylated anthocyanins—including Pn, Mv anthocyanins, and their derivatives—are relatively stable and represent the major pool of anthocyanins in mature berries [37]. We found the 73.8% and 10.8% increase of the proportion of Cy and Dp in T1 grapes compared to T2 ones, respectively, conversely, other monomer anthocyanins were lower. Presumably T1 treatment was not conducive to color stability and intensity of anthocyanins. In addition, the pH value can influence the color in young red wines, T2 and T3 treatments induced an anthocyanin increment. However, at the same time the high pH values (and of course the low total acidity levels, in particular in T2 treatment) induce a low red color intensity as a result of low levels of anthocyanins in flavylium cation form. In nature, anthocyanins are in equilibrium between the color flavylium cation and the colorless hydrated form. The equilibrium is driven to the red form when the pH of the wine decreases and to the no colored form when pH increases [36,38]. Thus, it is not only necessary to have an increase in anthocyanin content from a quantitative point of view.

In other fruits, Fe deficiency increased phenolic concentrations of peach [10], strawberry [39], and lemon fruits [12], but not in pear [10]. These results indicated the increase of phenolic concentrations in fruits may depending on the kinds of fruits. This prompted us to investigate the transcriptional regulation of the flavonoid pathway by Fe in grape berries.

### 2.3. Expression of the Flavonoid Pathway Genes

**Figure 2:**
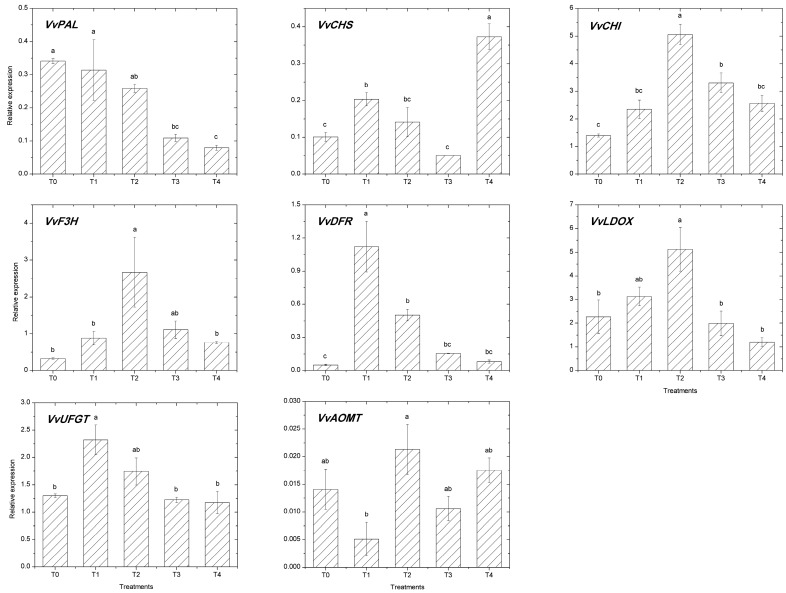
Transcript levels of genes in grape berries (mean ± sd; *n* = 3). Letters (a–c) indicate significant differences between treatments as calculated by Tukey’s HSD (*p* < 0.05). PAL, phenylalanine ammonia-lyase; CHS, chalcone synthase; CHI, chalcone isomerase; F3H, flavanone 3-hydroxylase; DFR, dihydroflavonol reductase; LDOX, leucoanthocyanidin dioxygenase; UFGT, UDP-glucose: flavonoid-3-*O*-glucosyltransferase; and AOMT, anthocyanin *O*-methyltransferase.

The expression of several structural genes (*VvPAL*, *VvCHS*, *VvCHI*, *VvF3H*, *VvDFR*, *VvLDOX, VvUFGT*, and *VvAOMT*) of the flavonoid pathway in grape berry skin were affected by iron fertilization (Figure 2). The gene of the early steps of this pathway *VvPAL* was upregulated in T0 and T1 as compared to T2 treatment, the transcript level was 1.3- and 1.2-fold higher in T0 and T1 treated berries, respectively, while the transcript level of T2 was higher than T3 and T4, and the change was 2.4- and 3.3-fold, respectively. Transcript level of *VvCHS* in T4 was significantly higher than other treatments, but there are not significant differences between the other four treatments. Compared to T2 treatment, the genes of *VvCHI* and *VvF3H* in the other four treatments were all inhibited, and the variation trends of the two genes were similar. The *VvDFR* transcript levels, which code for the dihydroflavonol-4-reductase, increase by 2.2-fold in the berries of the T1 treatment, compared to T2 treatment (Figure 2); conversely, the *VvDFR* in T0, T3, and T4 treatments was lower expressed compared to T2 treatment. In the late steps of anthocyanin biosynthesis, transcripts levels of, *VvLDOX* and *VvAOMT* were all upregulated in response to moderate Fe supply (T2), while the expression differences of these genes between the other four treatments were almost all insignificant. The UDP-glucose: flavonoid 3-*O*-glucosyltransferase (*VvUFGT*) transcript levels in T1 treatment grapes were more highly expressed compared to T2 treatment, but the difference was not significant.

The genes investigated in this study are key genes in the flavonoid pathway, which are usually used in related studies [17,22,23,24,25,26,27,28,29,30,31,32,33,34]. Phenylalanine ammonia-lyase (PAL) is a key enzyme for phenylpropanoid metabolism, it may indirectly related to anthocyanin biosynthesis [34]. In red grapes, PAL activity has two peaks, one during the early development of the fruit, and the other at maturity [23]. In the present study, the expression of *VvPAL* was upregulated with the treatments of lower Fe concentrations (T0 and T1) and downregulated with the treatment of higher Fe concentration (T3) compared to T2, and the differences of relative expression values were not significant. The high Fe concentration treatment (T4) decreased the value of gene expression of *VvPAL* significantly, this was possibly related to the obvious retarded grape ripening (Table 1).

The product naringenin chalcone of chalcone synthase (CHS) can be used to synthesize anthocyanins, proanthocyanidins, and other phenolic compounds [34]. Delgado et al. [21] reported that the tannin content of the berry skin decreased initially after veraison, then increased around the 28th day after veraison and reached the maximum amount a few days before ripening. In this study, the *VvCHS* relative expression of T4 treatment was significantly higher than other treatments. This is probably related to the berry growth stage of T4 treatment occuring a few days before ripening, which needed higher *VvCHS* expression to provoke higher amount of tannin. The difference of *VvCHS* relative expression of T0, T1, and T3 treatments was not significant compared to T2 treatment.

Chalcone isomerase (CHI) modified naringenin chalcone to its isomer naringenin flavanone which initially consists of the basic flavonoid skeleton (three rings of C6-C3-C6) [34], which is crucial to the subsequent reactions. Besides, flavanone 3-hydroxylase (F3H) can catalysis flavanones to produce the corresponding dihydroflavonols [34]. The two genes (*VvCHI* and *VvF3H*) identically responded to Fe treatments in this study. The total anthocyanins content of different Fe treatments were positively correlated with the two genes’ expression values. The higher expression of the two genes in T2 treatment may contribute to provide the potential substrates for anthocyanins biosynthesis. Consequently, total anthocyanins content of T2 treatment was significant higher than other treatments.

Dihydroflavonol reductase (DFR) can reduce dihydroflavonols to their corresponding leucoanthocyanidins, and the leucoanthocyanidins being used to synthesize the corresponding anthocyanidins by the catalysis of leucoanthocyanidin dioxygenase (LDOX). The LDOX can divide the whole flavonoid pathway into two parts: the basic flavonoid upstream pathway and the specific pathway for anthocyanin modification. In the specific pathway, the anthocyanidins are unstable and usually *O*-glycosylated at the C3 position by UDPglucose: flavonoid 3-*O*-glucosyltransferase (UFGT) using UDP-glucose as a sugar donor [23,34]. Then the methylation of the anthocyanins hydroxyl groups was under the catalysis of anthocyanin *O*-methyltransferase (AOMT). In this study, the expression values of *VvDFR* and *VvUFGT* in T1 treatment grape skins were higher than other treatments. This may provide more substrates for downstream pathway, and more substrates can be catalyzed to glycosylated anthocyanins. The expression value of *VvAOMT* in T1 treatment grape skins was lower than other treatments. This means that a smaller quantity of glycosylated anthocyanins be catalyzed to methylated anthocyanins. This may help to explain why the content of Cy and Dp in T1 treatment grapes were higher than those in other treatment grapes.

On the whole, compared to appropriate Fe treatment (T2), slight Fe deficiency (T1) treatment could upregulate genes *VvPAL*, *VvCHS*, *VvDFR*, and *VvUFGT*, but only gene *VvDFR* was showed significant difference in expression, while other treatments (T0, T3, and T4) were almost all downregulated the investigated genes except *VvPAL* in T0 grapes and *VvCHS* in T4 grapes. The effect on gene expression often depends not only on the treatment but also on the berry developmental stage. Therefore, changes in gene expression cannot be the only factors affecting the accumulation of anthocyanin compounds, which can be due to the complexity of the metabolic pathways. The total anthocyanin concentration of Fe deficiency and excess treatments grapes (T0, T1 and T3, T4) were lower than that of T2 treatment grapes, this was probably related not only to gene expression but also to the differences of sugar content of different treatments [18]. These results may suggest that anthocyanin accumulation was affected by Fe supply. However, whether the influence of Fe on anthocyanin level is a direct or indirect effect remains to be assessed by further experiment, taking into account the relationship between primary metabolism and secondary metabolism such as sugars and anthocyanins and related genes.

## 3. Materials and Methods

### 3.1. Plant Materials and Treatments

Four-year-old own-rooted vines of commercial wine grape cultivar Cabernet Sauvignon were transplanted into plastic pots (40 × 28 × 34 cm). Pots filled with a mixture of washed sand and perlite in a ratio of 3:1 (*v*/*v*) and watered with 1L of nutrient solution every three days. The basic nutrient solution had the following composition: (mM) 17 N, 4 P, 7 K, 5 Ca, 2.5 Mg, 2.5 S, (μM) 0.32 Cu, 6.72 Mn, 0.77 Zn, 46.25 B, and 0.5 Mo [40]. The nutrient solution was prepared from analytical grade chemicals; the Fe source used for evaluation was Fe-EDDHA (ferric ethylenediamine di (*o*-hydroxyphenylacetic) acid); with 6% Fe rather than as an inorganic iron in order to prevent precipitation. Five Fe concentrations (T0: 0, T1: 23, T2: 46, T3: 92, and T4: 184 μM) were used in the nutrient solution. There were three replicates per treatment in a randomized complete block design, each replicate consisting of three plants [41].

Treatments were carried out under greenhouse conditions from March to October 2015 at Northwest A&F University, Yangling, China (34.29° N, 108.35° E). The relative humidity was in the range 35%–85%, depending on the weather condition and time of the day and year. Temperature was controlled using fans during mid-season with the aim of keeping day temperature in the 20–35 °C range and night temperature above 15 °C. Grape berries from each replicate were harvested on September 14 (50 days after veraison), kept in foam boxes filled with ice bags, and taken to the laboratory within two hours. The skins of 100 grape berries were peeled and dried out with kitchen towel, then weighed and frozen in the liquid nitrogen and grinding. Grape skin powders were stored at −80 °C until use. The pulp was used to measure sugar, acid, and pH.

### 3.2. Reducing Sugar (RS) and Titratable Acid Content (TAC) Assay

Berry juice was obtained by hand pressing and used to determine RS, TAC, and pH. The contents of RS were measured according to the National Standard of the People’s Republic of China [42]. TAC was determined by adding 25 mL distilled water to 5 mL fresh extracted fruit juice. This mixture was automatically titrated with a solution of 0.05 mol·L^−1^ NaOH to a final pH of 8.2 (Mettler Toledo ET18 titrator; Mettler Toledo Instruments, Zurich, Switzerland). The results were expressed as grams of tartaric acid L^−1^ juice [24,39]. pH was measured with a pH meter (Mettler Toledo ET18 titrator; Mettler Toledo Instruments, Zurich, Switzerland).

### 3.3. Assay for Anthocyanins

#### 3.3.1. Extraction of Anthocaynins

The method of extraction of grape skin anthocyanins was used according to He et al. [32] with minor modification. Skin powder (3 g) was extracted in methanol solution (20 mL) containing 2% formic acid. This extraction was performed with the aid of ultrasonics for 10 min at 40% power and 25 °C, and then shaken in an orbital shaker (SHZ-88A, Taicang Experiment Equipment Factory, Taicang Jiangsu, China) at 25 °C for 30 min at a rate of 150 rpm. The homogenate was centrifuged at 8000 rpm for 10 min and the supernatant was collected. The residue was extracted four more times using the same procedure. Finally, all the supernatants were pooled into a distilling flask. Extracting agent was removed through a rotary evaporator (SENCO-R series, Shanghai Shensheng Biotech Co. Ltd., Shanghai, China) at 28 °C, then the residues were redissolved in 10 mL of high performance liquid chromatography (HPLC) mobile phase (see Section 3.3.2). All the steps were conducted in the dark. Three independent extractions were carried out for each treatment [28,32].

#### 3.3.2. HPLC-MS Analyses of Anthocyanins

The HPLC-MS analyses were carried out using an Agilent 1100 series LC-MSD trap VL, (Agilent Technologies, Santa Clara, CA, USA), equipped with a G1322A degasser, a G1311A QuatPump, a G1313A ALS, a G1316A Colcom, a G1315A DAD and a Kromasil 100–5 C18 column (250 × 4.6 mm, 5 μm). The mobile phases were: (A) formic acid/acetonitrile/water (2:6:92, *v*/*v*/*v*), and (B) formic acid/acetonitrile/water (2:54:44, *v*/*v*/*v*). The injection volume was 30 μL with a flow rate of 1.0 mL/min. The gradient elution of solvent B was applied as follows: 1–18 min, 10% to 25%; 18–20 min, 25%; 20–30 min, 25% to 40%; 30–35 min, 40% to 70% and 35–40 min, 70% to 100%. The column temperature was 50 °C and the detection wavelength was 525 nm. MS conditions were as follows: Electrospray ionization (ESI) interface, positive ion mode, 35 psi nebulizer pressure, 10 mL/min dry gas flow rate, 300 °C dry gas temperature, and scans at *m*/*z* 100–1000 [28,32,43].

#### 3.3.3. Qualitative and Quantitative Analysis of Anthocyanins

The anthocyanins were identified after comparison of elution order, retention time, and the weight of the molecular ion and the fragment ion with standards and references [28,29,31,32,43]. The quantification of anthocyanins was obtained by the use of external standards. The relative content of individual anthocyanins was obtained as the equivalent of malvidin-3-*O*-glucoside, using HPLC peak areas at the detection wavelength. The concentration of each anthocyanin was calculated on the basis of fresh weight of the whole berry, expressed as μg of malvidin-3-*O*-glucoside equivalents per g of grape berry [28,31,32,43].

### 3.4. RNA Extraction and Gene Expression Analysis

Total RNA of the grape berry skins was extracted using Universal Plant Total RNA Extraction Kit (BioTeke Co., Beijing, China). All RNA samples were digested to remove genomic DNA and reverse-transcribed in a 20 μL reaction mixture for cDNA synthesis using a EasyScript First-Strand cDNA Synthesis SuperMix Reagent Kit (TransBionovo Co., Beijing, China) as described in the manufacturer’s manuals. The integrity of RNA was verified by the existence of intact ribosomal bands following agarose gel electrophoresis. RNA purity was assessed based on absorbance ratio of 1.8 to 2.0 at A260/A280 nm using Nanodrop ND-1000 Spectrophotometer (Nanodrop Technologies, Rockland, DE, USA).

Relative expression of genes was measured by real-time PCR using SYBR Real-time PCR premixture (BioTeke Co.) on a Bio-Rad IQ5 Real-Time PCR system (Bio-Rad Laboratories, Berkeley, CA, USA) as described in the manufacturers’ manuals.

Real-time PCR reaction mixture (25 μL) was comprised of 12.5 μL 2 × Premix, 10.5 μL sterilized ddH_2_O, 1.0 μL cDNA, and 0.5 μL of each primer (10 μmol/L). The template cDNA was denatured at 95 °C for 30 s followed by 45 cycles of amplification at 95 °C for 15 s, 60 °C for 20 s, 72 °C for 30 s, and a melt cycle from 60 °C to 95 °C. The sequences of the primers used for real-time PCR were referred from the previous studies [17,44,45] or designed by Primer Premier 5 (Table S1). The transcript levels of each gene were normalized with *VvUbiquitin* as a reference gene [46]. All samples were measured in triplicate, every run included the *VvUbiquitin* control for each sample. The difference between the cycle threshold (Ct) of the target gene and the reference gene was used to obtain the normalized expression of the target gene, calculated as 2^−(Ct target − Ct *VvUbiquitin*)^ [17,46,47].

### 3.5. Statistical Analysis

Statistical analysis was performed by SPSS 20.0 software (IBM, Armonk, NY, USA). Tukey’s HSD test was used to assess statistically significant differences in the grape physical and chemical characteristics, the content of various anthocyanins and the transcription levels of genes between treatments. Figures were made by using the drawing software Origin 8.0 (OriginLab, Hampton, MA, USA).

## 4. Conclusions

In the present research, the effects of Fe supply on Cabernet Sauvignon grapes were studied. The results showed that moderate Fe supply produced higher RS, RS/TAC ratio, and concentration of anthocyanins, which contributed to the excellent performance of the grapes. Moreover, expression of anthocyanin biosynthetic pathway genes in grape skins was also influenced by Fe treatment which could help to explain in part the effect on individual anthocyanin concentrations.

## Figures and Tables

**Table 1:** Physical and chemical analyses of Cabernet Sauvignon berries and grape juice sampled from each iron supply treatment. RS = Reducing Sugar (expressed in gram equivalent glucose L^−1^), TAC = Titratable Acid Content (expressed in gram equivalent tartaric acid L^−1^). Different lowercase letters indicate significant differences between treatments as calculated by Tukey’s HSD test (*p* < 0.05).

Treatments	T0	T1	T2	T3	T4
RS	161.67 ± 0.85d	173.56 ± 0.49b	182.26 ± 0.54a	168.86 ± 0.46c	117.81 ± 0.22e
TAC	7.87 ± 0.09b	4.61 ± 0.05d	3.31 ± 0.02e	4.95 ± 0.06c	9.58 ± 0.11a
RS/TAC	20.55 ± 0.12d	37.63 ± 0.32b	55.03 ± 0.23a	34.10 ± 0.29c	12.30 ± 0.12e
pH	3.69 ± 0.04c	4.23 ± 0.02ab	4.35 ± 0.03a	4.11 ± 0.05b	3.45 ± 0.04d
Berry weight (g)	0.79 ± 0.04b	0.69 ± 0.02b	0.89 ± 0.02a	0.71 ± 0.02b	0.57 ± 0.03c
Skin/berry (%)	9.41 ± 0.22a	8.51 ± 0.59a	8.31 ± 0.44a	9.47 ± 0.29a	9.10 ± 0.08a

**Table 2:** Characterization of anthocyanin compounds in berry skins using HPLC-DAD-MS.

Peak	Retention Time (min)	Molecular Ion M^+^ (*m*/*z*)	Fragment Ion M (*m*/*z*)	Tentative Identification	Abbreviations Used	Ref.(s)
1	4.44	465	303	delphinidin-3-*O*-glucoside	Dp	[28,31]
2	6.27	449	287	cyanidin-3-*O*-glucoside	Cy	[28,31]
3	7.44	479	317	petunidin-3-*O*-glucoside	Pt	[28,31]
4	10.04	463	301	peonidin-3-*O*-glucoside	Pn	[28,31]
5	11.13	493	331	malvidin-3-*O*-glucoside	Mv	[28,31]
6	12.65	507	303, 465	delphinidin-3-*O*-(6-*O*-acetyl)-glucoside	Dp-acet	[28,31]
7	16.11	491	287, 449	cyanidin-3-*O*-(6-*O*-acetyl)-glucoside	Cy-acet	[28,31]
8	17.60	521	317, 479	petunidin-3-*O*-(6-*O*-acetyl)-glucoside	Pt-acet	[28,31]
9	18.52	611	303, 465	delphinidin-3-*O*-(6-*O*-coumaryl)-glucoside	Dp-coum	[28,31]
10	21.35	505	301, 463	peonidin-3-*O*-(6-*O*-acetyl)-glucoside	Pn-acet	[28,31]
11	22.32	535	331, 493	malvidin-3-*O*-(6-*O*-acetyl)-glucoside	Mv-acet	[28,31]
12	24.52	625	301, 463	peonidin-3-*O*-(6-*O*-caffeoyl)-glucoside	Pn-caff	[31]
13	25.01	595	287, 449	cyanidin-3-*O*-(6-*O*-coumaryl)-glucoside	Cy-coum	[28,31]
14	25.52	655	331, 493	malvidin-3-(6-*O*-caffeoyl)-glucoside	Mv-caff	[28,31]
15	26.30	625	317, 479	petunidin-3-*O*-(6-*O*-coumaryl)-glucoside	Pt-coum	[28,31]
16	27.51	609	301, 463	peonidin-3-*O*-(cis-6-*O*-coumaryl)-glucoside	cPn-coum	[28,32]
17	28.09	639	331, 493	Malvidin-3-*O*-(cis-6-*O*-coumaryl)-glucoside	cMv-coum	[28,32]
18	29.45	609	301, 463	peonidin-3-*O*-(*trans*-6-*O*-coumaryl)-glucoside	tPn-coum	[28,32]
19	29.98	639	331, 493	malvidin-3-*O*-(*trans*-6-*O*-coumaryl)-glucoside	tMv-coum	[28,32]

**Table 3:** Effects of iron supply on individual anthocyanins (μg/g berry) in grape berries. Different lowercase letters indicate significant differences between treatments as calculated by Tukey’s HSD test (*p* < 0.05).

Anthocyanins	T0	T1	T2	T3	T4
Dp	7.39 ± 0.23e	19.06 ± 0.30a	17.20 ± 0.04b	11.90 ± 0.02d	15.35 ± 0.02c
Cy	1.74 ± 0.006c	6.87 ± 0.002a	3.95 ± 0.030b	3.76 ± 0.853b	4.45 ± 0.012b
Pt	8.42 ± 0.027e	15.44 ± 0.006b	20.54 ± 0.002a	14.07 ± 0.078c	11.14 ± 0.012d
Pn	15.70 ± 0.02e	26.85 ± 0.13c	28.88 ± 0.05a	27.49 ± 0.18b	21.87 ± 0.02d
Mv	124.35 ± 0.23d	203.60 ± 0.36c	274.57 ± 0.40a	269.56 ± 0.31b	123.62 ± 0.10d
Dp-acet	2.95 ± 0.001d	6.23 ± 0.299b	8.11 ± 0.001a	5.34 ± 0.028c	5.09 ± 0.003c
Cy-acet	2.12 ± 0.001e	4.68 ± 0.253c	7.82 ± 0.019a	5.95 ± 0.009b	2.80 ± 0.010d
Pt-acet	4.65 ± 0.04e	7.86 ± 0.30c	14.17 ± 0.04a	8.92 ± 0.05b	5.60 ± 0.01d
Dp-coum	0.80 ± 0.001d	1.84 ± 0.151c	4.47 ± 0.048a	2.95 ± 0.317b	1.09 ± 0.024d
Pn-acet	7.87 ± 0.02e	13.68 ± 0.14c	18.85 ± 0.06a	14.69 ± 0.02b	10.86 ± 0.02d
Mv-acet	92.74 ± 0.37d	151.50 ± 0.69c	211.27 ± 0.11a	202.62 ± 1.53b	91.54 ± 0.15d
Pn-caff	0.38 ± 0.012c	0.82 ± 0.000b	2.42 ± 0.003a	1.00 ± 0.140b	0.13 ± 0.007c
Cy-coum	0.05 ± 0.005b	0.28 ± 0.002b	1.29 ± 0.023a	0.68 ± 0.499a,b	0.29 ± 0.016b
Mv-caff	2.43 ± 0.01d	4.02 ± 0.03c	7.28 ± 0.02a	4.39 ± 0.06b	1.38 ± 0.03e
Pt-coum	0.28 ± 0.005e	0.95 ± 0.009c	3.75 ± 0.010a	1.36 ± 0.034b	0.77 ± 0.019d
cPn-coum	0.14 ± 0.003d	0.39 ± 0.012c	1.94 ± 0.114a	0.90 ± 0.017b	0.35 ± 0.004c
tPn-coum	1.40 ± 0.03d	4.39 ± 0.14c	9.71 ± 0.07a	6.27 ± 0.02b	4.20 ± 0.02c
cMv-coum	0.72 ± 0.04d	1.20 ± 0.16c	4.99 ± 0.19a	1.90 ± 0.07b	0.94 ± 0.03c,d
tMv-coum	11.81 ± 0.08e	31.24 ± 0.26c	54.03 ± 0.22a	47.66 ± 0.09b	23.79 ± 0.24d
Total anthocyanins	285.95 ± 0.30e	500.90 ± 0.68c	695.24 ± 0.79a	631.43 ± 1.66b	325.25 ± 0.30d

## Supplementary Materials

Supplementary materials are available online.
