# Supplementary material for: Iron Supply Affects Anthocyanin Content and Related Gene Expression in Berries of Vitis vinifera cv. Cabernet Sauvignon
Source: Molecules. 2017 Feb 14;22(2):283. doi: 10.3390/molecules22020283 (PMC6155850; doi:10.3390/molecules22020283)
Supplement: Supplementary file 1 [file molecules-22-00283-s001.pdf]

Supplementary Materials: Iron Supply Affects Anthocyanin Content and Related Gene Expression in Berries of *Vitis vinifera* cv. Cabernet Sauvignon

Pengbao Shi, Bing Li, Haiju Chen, Changzheng Song, Jiangfei Meng, Zhumei Xi and Zhenwen Zhang

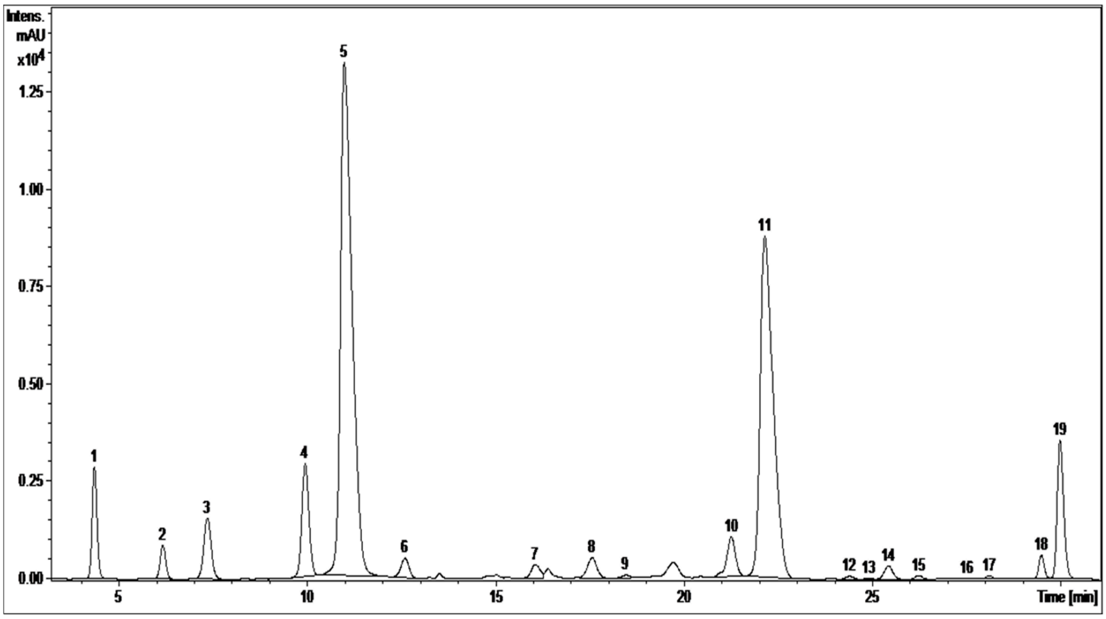

Figure S1. Chromatography of anthocyanins in the grape skins. Peak numbers correspond to the labeling adopted in Table 2.

Table S1. Primers used for quantification of transcripts by real time quantitative PCR.

Gene ID	Gene Name	Primer	Sequence	Reference
XM_003633937	*VvPAL*	F	CCGAGCATCAACTAAATCCA	This study
		R	GGCAGAGTGCCACTAGGTAT	
AB066274	*VvCHS*	F	GGTTTTGGACCAGGCTTGACT	[45]
		R	GAGATAAATACCTTACTCCTATTCAAC	
NM_001281104.1	*VvCHI*	F	AGACTGTGGAGGAGTTAGCG	This study
		R	GAATGGAGTTGCCTGGTG	
NM_001281105.1	*VvF3H*	F	CCTACCCACTACGAACCAG	This study
		R	CTTGAACCTCCCATTGCT	
X75964	*VvDFR*	F	GAAACCTGTAGATGGCAGGA	[44]
		R	GGCCAAATCAAACTACCAGA	
X75966	*VvLDOX*	F	AGGGAAGGGAAAACAAGTAG	[44]
		R	ACTCTTTGGGGATTGACTGG	
AF000372	*VvUFGT*	F	GGGATGGTGATGGCTGTGG	[44]
		R	ACATGGGTGGAGAGTGAGTT	
GU237132.1	*VvAOMT*	F	CATTGCCGATGGGAAAGAAGAAGG	[17]
		R	CGTAAGCGATTATGCCTCCAACTC	
XM_002273532	*VvUBIQUITIN*	F	GTGGTATTATTGAGCCATCCTT	[46]
		R	AACCTCCAATCCAGTCATCTAC	
